# Inflammatory macrophages facilitate mechanical stress-induced osteogenesis

**DOI:** 10.18632/aging.102833

**Published:** 2020-02-25

**Authors:** Fan Zhang, Le Huan, Tao Xu, Guozheng Li, Bing Zheng, Hong Zhao, Yongfei Guo, Jiangang Shi, Jingchuan Sun, Aimin Chen

**Affiliations:** 1 Department of Orthopedic Surgery, Changzheng Hospital, Second Military Medical University, Shanghai 200001, China; 2 Department of Orthopedic Surgery, No. 906 Hospital of the People’s Liberation Army, Ningbo 330212, China; 3 Department of Spine Surgery, LinZhou Hospital of Traditional Chinese Medicine, Linzhou 456550, China

**Keywords:** macrophages, mechanical stress, distraction osteogenesis (DO), saporin-CD11b

## Abstract

Mechanical stress has been recognized as a key inducer of bone regeneration in bone damage, which is experimentally mimicked by distraction osteogenesis (DO), a bone-regenerative process induced by post-osteotomy distraction of the surrounding vascularized bone segments, and realized by new bone formation within the distraction gap. The mechanisms that underlie the DO-induced bone regeneration remain poorly understood and a role of macrophages in the process has been inadequately studied. Here, in a mouse model of DO, we showed significant increase in macrophages in the regeneration area. Moreover, in a loss-of-function approach by depleting inflammatory macrophages, the bone regeneration was compromised by assessment of histology and molecular biology. Thus, our study demonstrates the necessary participation of inflammatory macrophages in the process of DO-induced bone regeneration, and suggests that targeting inflammatory macrophages may help to improve clinical bone repair.

## INTRODUCTION

All accidents, bone tumor resection and debridement of bone infections may cause complex fractures [1]. In such situation, the bone regeneration occurs to compromise the loss of bone tissue and to connect the broken bones [2]. When the bone loss exceeds a certain range, the bone fails to repair the defect completely, and requires therapeutic approaches to restore [3].

Mechanical stress has been recognized as a key inducer of bone regeneration in bone damage, which is experimentally mimicked by distraction osteogenesis (DO), a bone-regenerative process induced by post-osteotomy distraction of the surrounding vascularized bone segments, and realized by new bone formation within the distraction gap [4].

DO is efficacious for reconstructing bony defects, whereas the underlying molecular biology and biology of bone development remain poorly understood [5]. So far, it is believed that mechanical stimulation by DO induces a biological response of new bone regeneration that is accomplished by a cascade of biologic processes including differentiation of pluripotential cells, tissue angiogenesis and mineralization, as well as remodeling in the damaged region [6]. The most important remaining question is how the distraction-induced mechanical forces are translated into biologic signals to induce new bone regeneration.

Severe inflammation occurs during bone fraction, which is further enhanced by DO [7]. Thus, it is understandable that inflammation could play a critical roke in the bone regeneration induced by DO [8–10]. In this regard, innate immunity within bone damage regions could be mediated by activated and inflammatory macrophages [11]. Since macrophages have been found to play a variety role in tissue repair and regeneration [12–14], it is noteworthy to study the effects of inflammatory macrophages on DO-induced bone regeneration and repair. However, to our surprise, such studies are very lacking currently [15].

Here, in a mouse model of DO, we showed significant increase in macrophages in the regeneration area. Moreover, in a loss-of-function approach by depleting inflammatory macrophages, the bone regeneration was compromised by assessment of histology and molecular biology.

## RESULTS

### Analysis of biopsy tissue during DO

**Figure 1:**
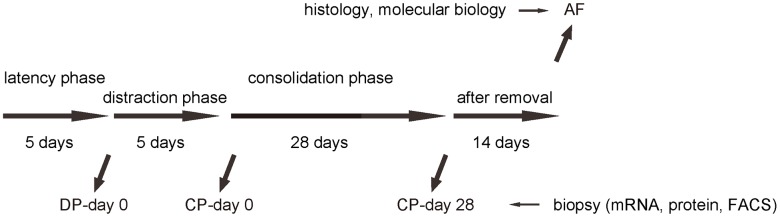
**Analysis of biopsy tissue during DO.** Illustration of the model: We performed DO of the lower limb on the C57/Bl6 mice. After surgery, there was a 5 days’ latency for the mice the recover. Afterwards, there was a 5 days’ distraction phase constituted by a rate of 0.5 mm’ distraction per 24 hours. After distraction phase, a 28 days’ consolidation phase was applied, followed by removal of external fixers and a 14 days’ delay for final analysis. In order to analyze macrophages during DO-induced bone regeneration, we took biopsy of the tissue in the surgical/regeneration area at day 0 of distraction phase (DP-day 0), day 0 of consolidation phase (CP-day 0) and day 28 or consolidation phase (CP-day 28).

We performed DO of the lower limb on the C57/Bl6 mice. After surgery, there was a 5 days’ latency for the mice the recover. Afterwards, there was a 5 days’ distraction phase constituted by a rate of 0.5 mm’ distraction per 24 hours. After distraction phase, a 28 days’ consolidation phase was applied, followed by removal of external fixers and a 14 days’ delay for final analysis (Figure 1). In order to analyze macrophages during DO-induced bone regeneration, we took biopsy of the tissue in the surgical/regeneration area at day 0 of distraction phase (DP-day 0), day 0 of consolidation phase (CP-day 0) and day 28 or consolidation phase (CP-day 28). CD68 is a specific cell surface marker for macrophages. The tissue was analyzed for CD68 mRNA by RT-qPCR, for CD68 protein by ELISA and for CD68+ cells by Fluorescence-activated cell sorting (FACS) (Figure 1).

### Macrophages increase during DO

**Figure 2:**
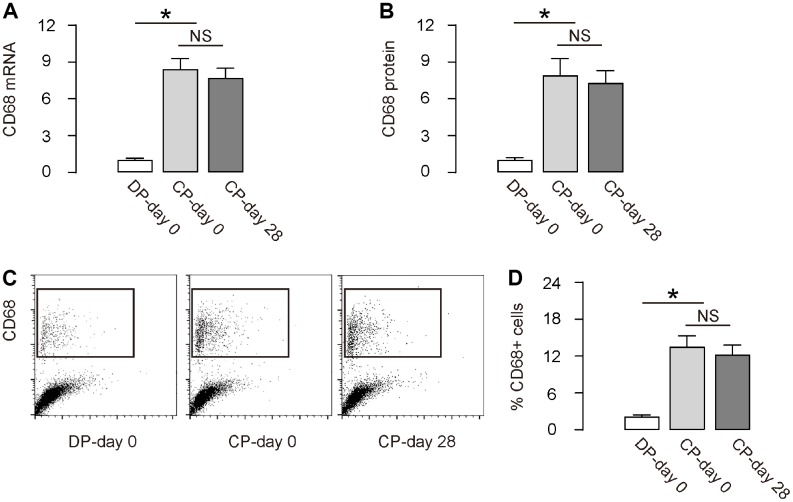
**Macrophages increase during DO.** (**A**, **B**) RT-qPCR for CD68 mRNA (**A**) and ELISA for CD68 protein (**B**) in regenerating bone tissue. (**C**, **D**) FACS for CD68+ cells in regenerating bone tissue by representative flow charts (**C**), and by quantification (**D**). DP-day 0: day 0 of distraction phase, CP-day 0: day 0 of consolidation phase, CP-day 28: day 28 or consolidation phase. *p<0.05. NS: non-significant. N=8.

We detected significantly increased CD68 mRNA (Figure 2A) and CD68 protein (Figure 2B) during DO (CP-day 0 versus DP-day 0). Moreover, the increase CD68 mRNA and protein levels did not decrease at the end of consolidation phase (CP-day 28 versus CP-day 0, Figure 2A, 2B). Furthermore, flow cytometry analysis exhibited similar results, showing significantly increased CD68+ cells in the total tissue cells in the regeneration region, by representative flow charts (Figure 2C), and by quantification (Figure 2D). Together, these data suggest that inflammatory macrophages increase during DO.

### Analysis of biopsy tissue during DO with macrophage depletion

**Figure 3:**
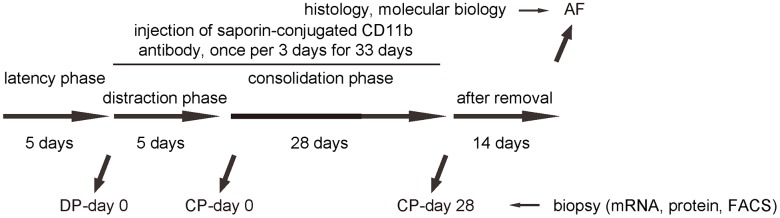
**Analysis of biopsy tissue during DO with macrophage depletion.** Illustration of the model: We performed DO of the lower limb on the C57/Bl6 mice. After surgery, there was a 5 days’ latency for the mice the recover. Afterwards, there was a 5 days’ distraction phase constituted by a rate of 0.5 mm’ distraction per 24 hours. After distraction phase, a 28 days’ consolidation phase was applied, followed by removal of external fixers and a 14 days’ delay for final analysis. In order to analyze macrophages during DO-induced bone regeneration, we took biopsy of the tissue in the surgical/regeneration area at day 0 of distraction phase (DP-day 0), day 0 of consolidation phase (CP-day 0) and day 28 or consolidation phase (CP-day 28). In order to understand the role and necessity of macrophages in DO-induced bone regeneration, we performed an interference by inducing macrophage depletion during DO. DO-surgery-treated mice received i.v. injection of either saporin-CD11b once every 3 days, or control rat IgG of same frequency (IgG). The injection started at DP-day 0 and ended at CP-day 28.

In order to understand the role and necessity of macrophages in DO-induced bone regeneration, we performed an interference by inducing macrophage depletion during DO. Saporin is a protein also called as ribosome inactivating protein (RIP) due to its N-glycosidase activity from the seeds of Saponaria officinalis. Saporin contains some toxic molecules, including ricin and abrin which are able to enzymatically inactivate the ribosomes of the cells to shut down protein synthesis and cause cell death [16]. A saporin-conjugation to CD11b antibody (specifically against macrophages) causes specific macrophage death and depletion [16]. DO-surgery-treated mice received i.v. injection of either saporin-CD11b once every 3 days [16], or control rat IgG of same frequency (IgG). The injection started at DP-day 0 and ended at CP-day 28 (Figure 3).

### Saporin-CD11b reduces macrophages increase during DO

**Figure 4:**
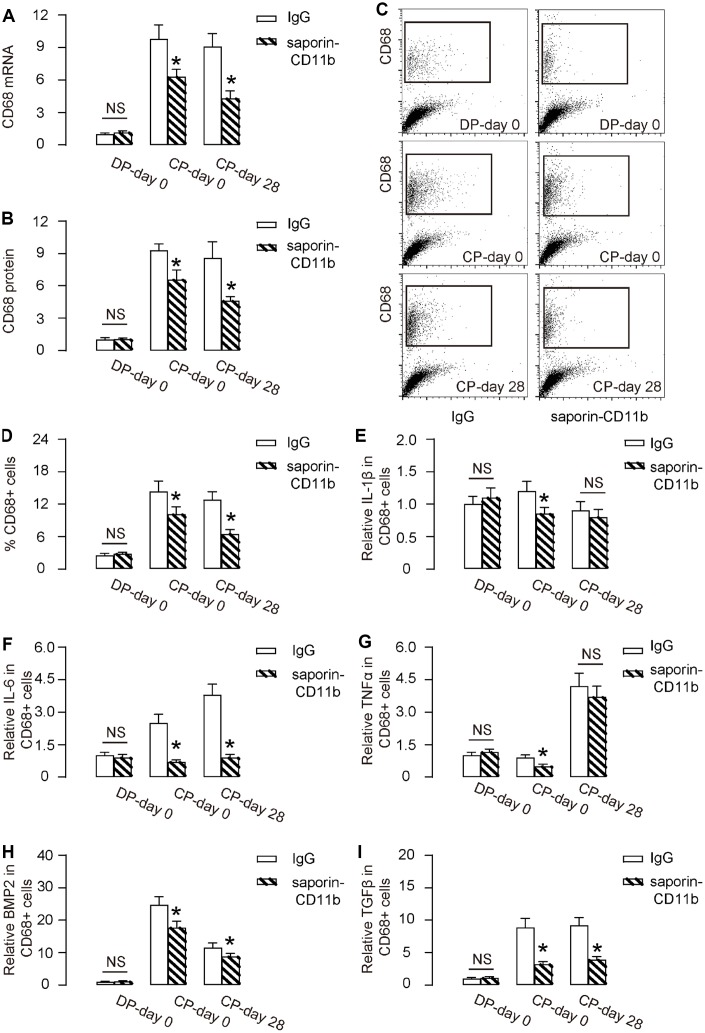
**Saporin-CD11b reduces macrophages increase during DO.** (**A**, **B**) RT-qPCR for CD68 mRNA (**A**) and ELISA for CD68 protein (**B**) in regenerating bone tissue from saporin-CD11b-treated mice or control IgG-treated mice. (**C**, **D**) FACS for CD68+ cells in regenerating bone tissue saporin-CD11b-treated mice or control IgG-treated mice by representative flow charts (**C**), and by quantification (**D**). (**E**–**I**) ELISA for IL-1β (**E**), IL-6 (**F**), TNFα (**G**), BMP2 (**H**) and TGFβ (**I**) from FAC-sorted CD68+ macrophages. DP-day 0: day 0 of distraction phase, CP-day 0: day 0 of consolidation phase, CP-day 28: day 28 or consolidation phase. *p<0.05. NS: non-significant. N=8.

By biopsy, we detected significantly reduced CD68 mRNA (Figure 4A) and CD68 protein (Figure 4B) in tissue from saporin-CD11b-treated mice during DO (CP-day 0 versus DP-day 0, or CP-day 28 versus DP-day 0,). Moreover, the reduction in CD68 mRNA and protein levels appeared to be more pronounced at the end of consolidation phase (CP-day 28 versus CP-day 0, Figure 4A, 4B). Furthermore, flow cytometry analysis exhibited similar results, showing significantly reduction in CD68+ cells in the total tissue cells in the regeneration region of saporin-CD11b-treated mice, by representative flow charts (Figure 4C), and by quantification (Figure 4D). Key cytokines (IL-1β, IL-6, TNFα, BMP2 and TGFβ) that are involved in DO were analyzed in purified CD68+ macrophages (Figure 4E–4I), showing significant reduction of IL-6 (Figure 4F), BMP2 (Figure 4H) and TGFβ (Figure 4I) at DP-day 0 and CP-day 28 from saporin-CD11b-treated mice, than those from control rat IgG-treated mice. Since macrophages are a major source of these cytokines at the determined time point during DO, these data together suggest that saporin-CD11b reduces macrophages increase during DO.

### Macrophage depletion compromises DO-induced bone regeneration

**Figure 5:**
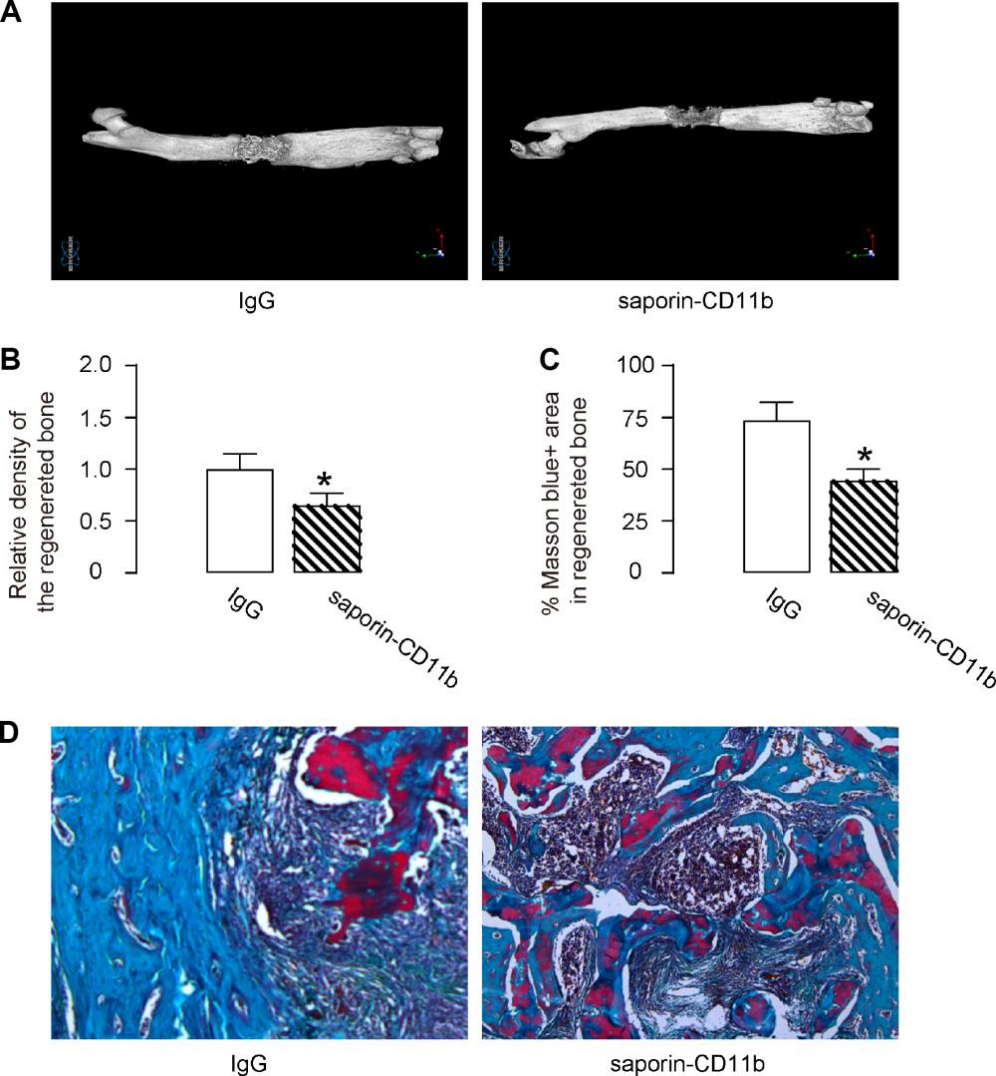
**Macrophage depletion compromises DO-induced bone regeneration.** Morphological histological analysis of the bone specimens was performed 14 days after removal of external fixers by micro-CT. (**A**, **B**) The density of the regenerative bone was assessed, shown by representative images (**A**), and by quantification (**B**). (**C**, **D**) Masson staining was also performed, shown by quantification (**C**), and by representative images (**D**). *p<0.05. NS: non-significant. N=8.

Morphological histological analysis of the bone specimens was performed 14 days after removal of external fixers by micro-CT. The density of the regenerative bone was assessed, showing that macrophage depletion by saporin-CD11b significantly reduced the density of the regenerative bone, shown by representative images (Figure 5A), and by quantification (Figure 5B). Masson staining was also performed, showing that macrophage depletion by saporin-CD11b significantly reduced the blue-stained area (representing collagen and bone) in the regenerated bone, shown by quantification (Figure 5C), and by representative images (Figure 5D). Hence, macrophage depletion compromises DO-induced bone regeneration.

### Macrophage depletion decreases regeneration-associated proteins

**Figure 6:**
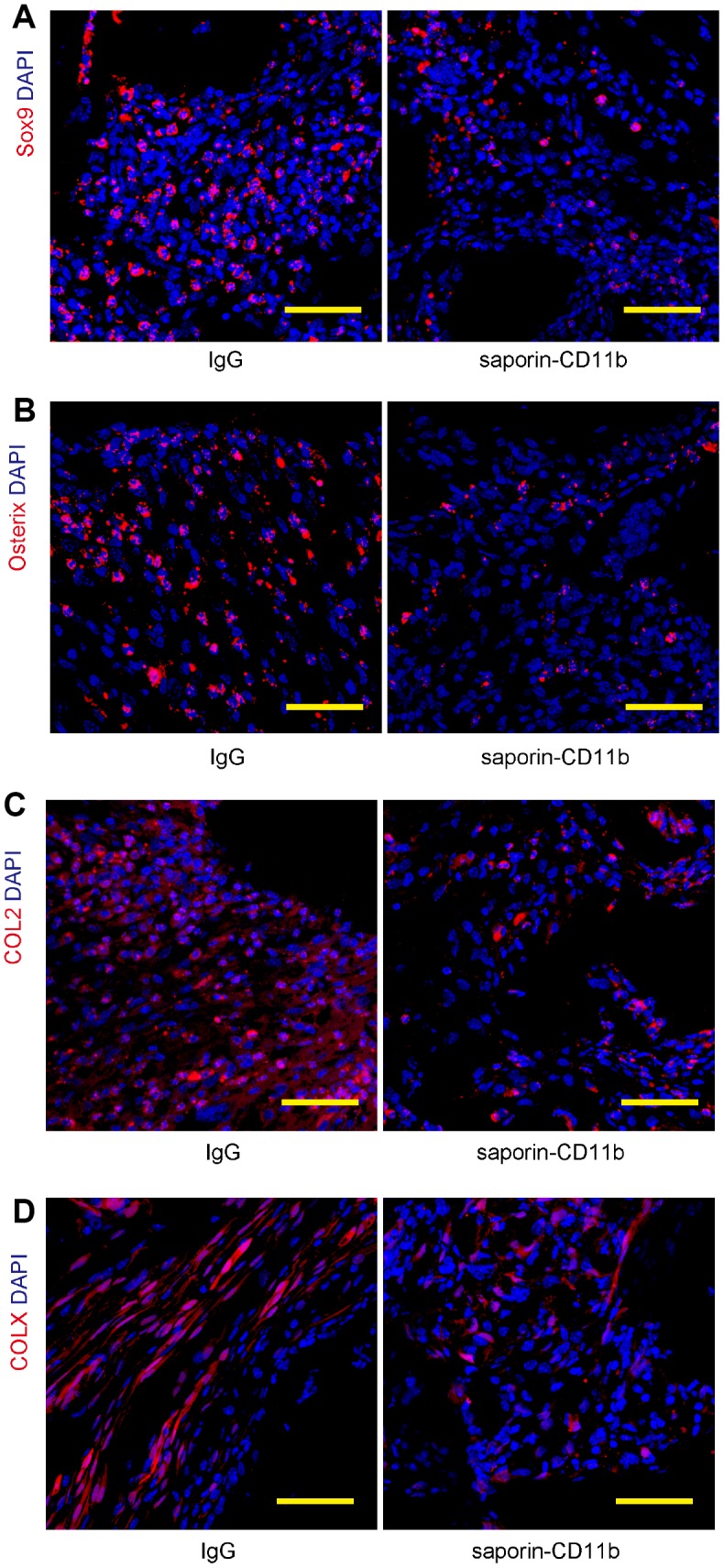
**Macrophage depletion decreases regeneration-associated proteins.** (**A**–**D**) Regeneration of the bone was assessed by examination of 4 regenerative markers, Sox9 (**A**) Osterix (**B**), COL2 (**C**) and COL X (**D**), by immunohistochemistry. N=8. Scale bars are 50μM.

Many of the signal transduction pathways regulating the progression of mesenchymal condensations to bone and cartilage have been found recapitulated in fracture healing. Among the key factors, Sox9 is a master regulatory transcription factor for osteogenic and chondrogenic specification, while Osterix is a transcription factor essential for osteoblast differentiation and bone mineralization. The expression of essential cartilage-related collagen genes including Collagen II (COL2) and Collagen X (COL X) generates an extracellular collagen matrix during bone regeneration. Hence, the bone regeneration was finally assessed by examination of these regeneration markers by immunohistochemistry (Figure 6A–6D). Our data showed that macrophage depletion by saporin-CD11b significantly reduced the Sox9, Osterix, COL2 and COL X levels in the regenerated bone (Figure 6A–6D).

## DISCUSSION

Bone regenerative capacity is limited, and is unable to repair large bone defects through its own regeneration. Tissue engineer is thus required to repair large bone defects. At present, ideal technology of tissue engineering bone is lacking, and repairing the bone defect using tissue engineer is still under investigation. Tissue engineering requires 3 necessary components: a progenitor or stem cell to produce the injured tissue, growth factors to provide the necessary inductive signals to facilitate the regeneration process, and a scaffold to guide three-dimensional configuration of tissue remodeling [17]. Clinical use of DO is essentially a form of bone tissue engineering.

Although the previous studies have made great effort to understand the molecular mechanisms that underlie the DO-induced bone regeneration, the most important question how the distraction-induced mechanical forces are translated into biologic signals to induce new bone regeneration remains unclear. Indeed, during DO, the bone-anchored distractor device provides a rigid space mimicking a scaffold. Progenitor cells are conveniently provided by the niche surrounding the distraction site. However, the growth factors that support and promote the bone regeneration are not well determined [17].

Since the injured/regenerating niche constitutes an inflammatory environment that produces a variety of cytokines and inflammatory cytokines to affect the regenerating progress, and these inflammatory factors may mediate the differentiation of stem or progenitor cells into chondrocytes and osteoblasts [18], we hypothesized that macrophages may play a non-redundant role in the process. Macrophages are important inflammatory cells involved in immunity. Recent researches have revealed multiple functions of macrophages that includes not only classical phagocytic and pro-inflammatory effects but also mediating wound healing and tissue-remodeling [12–14]. Indeed, here with the aid of a specific macrophage depletion approach, we found that macrophages are necessary for DO-induced bone regeneration. Impairment of macrophages in the regeneration niche may reduce the required growth factors that are necessary for bone regeneration, and these growth factors are believed to be mainly produced and secreted by macrophages [12–14]. From FAC-purified macrophages, we detected significant decreases in IL-6, BMP2 and TGFβ at DP-day 0 and CP-day 28 from saporin-CD11b-treated mice than those from control rat IgG-treated mice. It is noteworthy that BMP2 and TGFβ are cytokines predominantly produced by alternatively activated macrophages with important during tissue regeneration and remodeling, while IL-6 is a cytokine with complicated functions for both pro-inflammation and anti-inflammation [19]. Since macrophages are the major source of these cytokines at these time points, the reduction in IL-6, BMP2 and TGFβ from saporin-CD11b-treated mice confirmed the efficiency of macrophage depletion. On the other hand, the pre-inflammatory cytokines IL-1β and TNFα were not dramatically decreased, likely due to that at the examined time points, classical phagocytotic macrophages that produced these pre-inflammatory cytokines were already replaced by alternative macrophages.

Bone regeneration primarily occurs through endochondral bone formation, in which mesenchymal condensations determine the generation and proliferation scale of a bone. The transcription factor Sox9 is specifically expressed in progenitors for osteochondrocytes in the mesenchymal condensations. The absence of Sox9 in mice results in a complete defect of bone formation. Osterix is essential for the coupling of terminal cartilage differentiation and endochondral ossification in mandibular condylar cartilage [20]. Major cartilaginous matrix proteins COL2 [21] and COL X [22] were then activated. This impairment of bone regeneration in this study was not only confirmed by histology, but also mechanistically by analysis on these regenration-associated factors.

To summarize, here we demonstrate a critical role of macrophages in the bene regeneration induced by DO. Given the complex and dynamic phenotypes and functions of macrophages, it is important to further study the interactive involvement of macrophages in the DO-induced bone regeneration.

## MATERIALS AND METHODS

### Protocol approval

All mouse experiments were approved by the Institutional Animal Care and Use Committee at the Second Military Medical University (Animal Welfare Assurance). Mice were housed in Pathogen-free environment. C57BL/6 mice were purchased from Joint Ventures Sipper BK Experimental Animal (Shanghai, China). The 12-week-old female mice were used for the experiments.

### The animal manipulations

DO of the lower limb of the mice was generated. In brief, an anterior longitudinal incision was made on the right lower leg under intraperitoneal anesthesia with 2.5% Isoflurane (Sigma-Aldrich, Shanghai, China). After finalization of fibulotomy, a 26-gauge needles were inserted at both ends of the tibia and then were fixed with the external fixator consisted of two incomplete acrylic resin rings and an expansion screw. After completion of polymerization, osteotomy was performed at the middle of the diaphysis in the tibia. The wound was closed with a 4–0 nylon suture. The DO protocol was consisted of 5 days of latency period followed by 5 days’ distraction phase constituted by a rate of 0.5 mm’ distraction per 24 hours. After distraction phase, a 28 days’ consolidiation phase was applied, followed by removal of external fixers and a 14 days’ delay for final analysis.

For saporin-mediated depletion of macrophages, DO-surgery-treated mice received i.v. injection of either saporin-conjugated antibody against the macrophage surface marker CD11b (saporin-CD11b; 20μg; Advanced Targeting Systems, San Diego, CA, USA) once every 3 days [16], or control rat IgG of same frequency (IgG). The injection started at day 0 at distraction phase and ended at the 28^th^ day of consolidation phase.

### Micro-CT analysis

Bone specimens were fixed in paraformaldehyde for 48 hours, after which MicroCT examination was applied.

### Quantitative RT-PCR

The quantitative RT-PCR assay is summarized as follows. Total RNA was extracted by total RNA extraction kit (Qiagen, Valencia, CA, USA) according to instructions. Total RNA is transcribed by reverse transcription kit (Qiagen). The primers used for RT-qPCR were: GAPDH (sense: AGGGCTGCTTTTAACTCTGGT, anti-sense: GGCATGGACTGTGGTCATGAG); CD68 (sense: ACTTCGGGCCATGTTTCTCT, antisense: GCTGGTAGGTTGATTGTCGT); The average of three cycles was used to calculate gene expression, with GAPDH as an internal control.

### ELISA

Mouse CD68 protein was determined by an ELISA kit (MBS923382, Mybiosource Inc, San Diego, CA, USA). Mouse IL-1β, IL-6, TNFα, TGFβ protein were determined by corresponding ELISA kits (DY401, M6000B, MTA00B, MB11B, R&D, Los Angeles, CA, USA). Mouse BMP2 protein was determined by an ELISA kit (ab119582, Abcam, Cambridge, MA, USA).

### Histology and immunohistochemistry

After the sample was fixed in paraformaldehyde for 48 hours, and then prepared into 4um-thickness consecutive sections. Masson-trichrome staining was performed using a Trichrome Stain Kit (Sigma-Aldrich). For immunohistochemistry, sections were incubated with goat polyclonal anti-COL2 (ab34712, Abcam) or rabbit polyclonal anti-COL X (ab58632, Abcam) or mouse monoclonal anti-Sox9 (sc-166505, Santa Cruz Biotechnology, Dallas, TX, USA) or rabbit polyclonal anti-Osterix (ab94744, Abcam) overnight at 4 °C, followed by anti-goat, anti-mouse or anti-rabbit secondary antibodies (Jackson ImmunoResearch Labs, West Grove, PA, USA), correspondingly. Primary antibody and secondary antibody dilution ratio was 1:100. DNA were stained by DAPI (Sigma-Aldrich). The images were obtained by a laser confocal microscope.

### Statistical analysis

All values represent the mean ± standard deviation (SD). Statistical analysis of group differences was carried out using a one-way analysis of variance (ANOVA) test, followed by the Fisher’s Exact Test to compare two groups (GraphPad Prism 6.0, GraphPad Software, Inc. La Jolla, CA, USA). A value of p<0.05 was considered statistically significant.
